# Protein disulfide isomerase a multifunctional protein with multiple physiological roles

**DOI:** 10.3389/fchem.2014.00070

**Published:** 2014-08-26

**Authors:** Hyder Ali Khan, Bulent Mutus

**Affiliations:** Chemistry and Biochemistry Department, University of WindsorWindsor, ON, Canada

**Keywords:** PDI, protein disulfide isomerase, chaperone, oxidoreductase, disulfides, endoplasmic reticulum, cell surface

## Abstract

Protein disulfide isomerase (PDI), is a member of the thioredoxin superfamily of redox proteins. PDI has three catalytic activities including, thiol-disulfide oxireductase, disulfide isomerase and redox-dependent chaperone. Originally, PDI was identified in the lumen of the endoplasmic reticulum and subsequently detected at additional locations, such as cell surfaces and the cytosol. This review will provide an overview of the recent advances in relating the structural features of PDI to its multiple catalytic roles as well as its physiological and pathophysiological functions related to redox regulation and protein folding.

## Introduction

In 1963, microsomal preparations of rat liver were shown to reactivate reduced ribonuclease A (Goldberger et al., 1963). The enzyme that catalyzed this reaction was eventually identified as protein disulfide isomerase (PDI; EC 5.3.4.1). PDI is one of 20 proteins belonging to the PDI family (Kozlov et al., 2010). The proteins in this family all contain at least one domain with a thioredoxin-like fold(βαβαβαββα), but may vary in length and domain arrangement (Kozlov et al., 2010).

**Figure 1:**
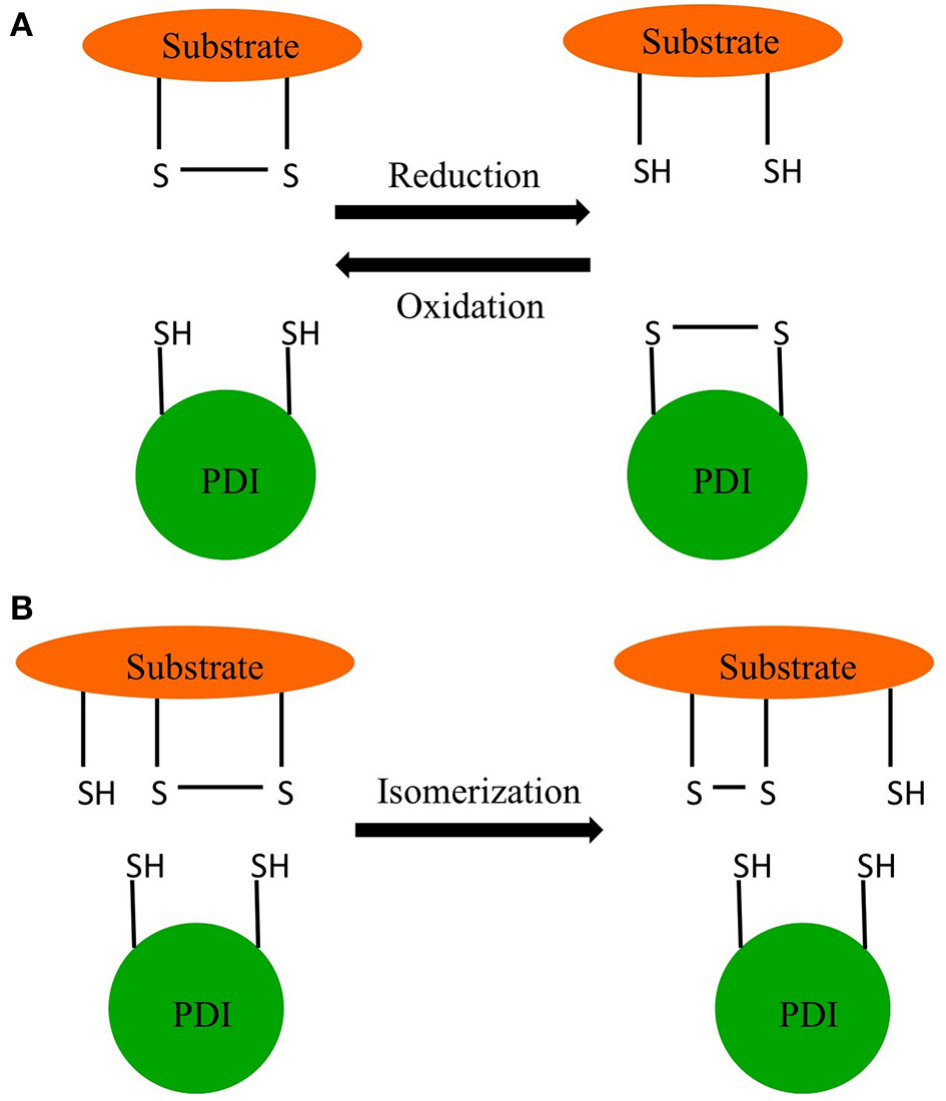
**Redox reactions of PDI. (A)** Oxidation and reduction. **(B)** Isomerization.

Edman and coworkers were able to determine the active site sequence of rat PDI as WCGHCK through sequencing of cDNA (Edman et al., 1985). This sequence was homologous to the active site of thioredoxin and was found to catalyze the reduction and isomerization of disulfide bonds and the oxidation of thiols (Figure 1) (Holmgren, 1968). In 1994 Cai and coworkers discovered PDI also had chaperone activity in addition to its redox activity (Cai et al., 1994). In 1998, McLaughlin and Bulleid showed that the chaperone activity was independent of the redox status of active site thiols (McLaughlin and Bulleid, 1998). The three catalytic activities of PDI, thiol redox, disulfide exchange, and chaperone are central to endoplasmic reticulum (ER) function (Maattanen et al., 2010).

PDI is expressed in almost all mammalian tissues (Marcus et al., 1996; Noiva, 1999). Although PDI has a C-terminal KDEL ER retention sequence, significant amounts of this protein were shown to escape the ER and were detected in the nucleus, cytosol, cell surface, and extracellularly (Edman et al., 1985; Koch, 1987; Yoshimori et al., 1990).

This review will focus on recent discoveries on PDI structure-function and physiological and pathophysiological roles arising from its localization in tissues including hemostasis, facilitation of pathogen entry, and reactive nitrogen and oxygen signaling. For more information on the role PDI in cancer, lipid homeostasis, infertility etc., the readers are directed to the reviews by Bulleid and Ellgaard (2011), Andreu et al. (2012), Laurindo et al. (2012), and Benham (2012).

## PDI structure

**Figure 2:**
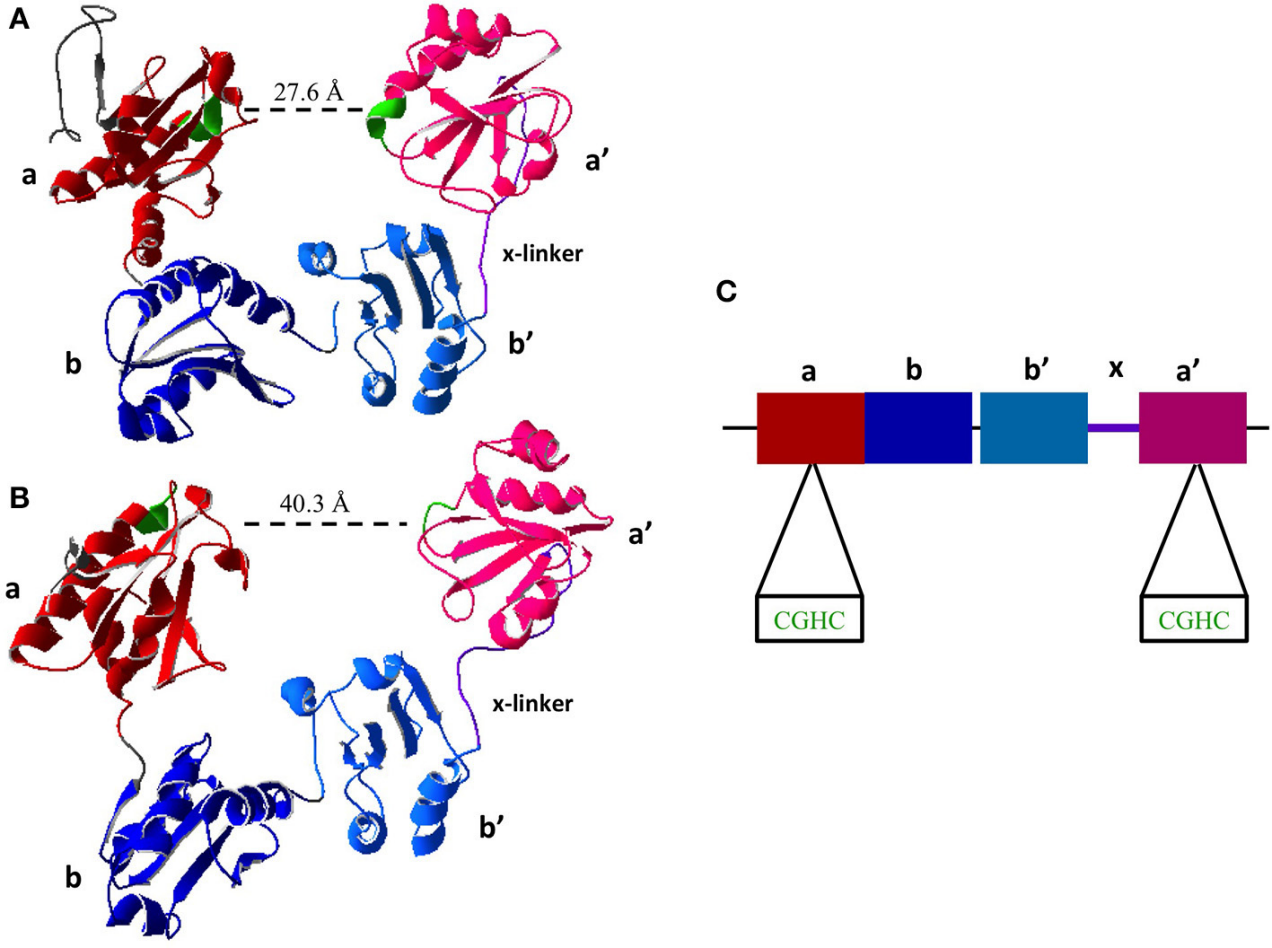
**Crystal Structure of Reduced and Oxidized PDI. (A)** Reduced crystal structure of human PDI (PDB ID: 4EKZ). **(B)** Oxidized crystal structure of human PDI (PDB ID: 4EL1). **(C)** Schematic of PDI domains present in the crystal structure, as well as the two active site CXXC motifs (Crystal structures created using Swiss PDBViewer) (Wang et al., 2013).

All members of the PDI family share the thioredoxin-like domain structure characterized by the βαβαβαββα fold (Kemmink et al., 1997). PDI contains four thioredoxin-like domains ***abb'a'***. The two redox active sites containing the CXXC motif are found in the ***a*** and ***a'*** domains (Figure 2). The active site domains are linked by the ***b*** and ***b'*** domains. There is also a small interdomain region known as the **x**-linker located between ***b'*** and ***a'*** (Freedman et al., 1998; Alanen et al., 2003). For an in depth review on the structure of PDI the reader is directed to a recent review by Kozlov et al. (2010).

Recent advances in NMR and x-ray crystallography have given further insight into PDI structure and function, by identifying the ***b'*** as the chaperone domain. Through NMR, the structure and amino acid residues of the ***b'*** domain were observed to interact with unfolded RNase A, an oft used enzyme to assay the chaperone activity of PDI. The ***b'*** domain contains a large multivalent hydrophobic surface allowing for a structurally promiscuous binding site (Denisov et al., 2009). In addition, computational analysis indicates that the ***bb'*** domains contain 4 cavities allowing for the possible binding of a variety of ligands (see Section PDI Chaperone Activity). Recently human PDI is observed to dimerize *in vivo* through the binding of ***bb'*** (Bastos-Aristizabal et al., 2014).

## PDI chaperone activity

The chaperone activity of PDI is an essential area of study to further understand the protein folding related roles of PDI in the ER as well as neuronal tissues (see Section PDI and Coagulation). Historically the chaperone activity of PDI was assessed by a variety of methods depending on renaturation of denatured proteins monitored by activity-gain or loss-of-aggregates (Shao et al., 2000; Ben Khalaf et al., 2012; Wang et al., 2012; Hashimoto and Imaoka, 2013). A recent addition to this list utilizes acid-denatured green fluorescent protein (GFP), which interacts with a chaperone protein like PDI, and refold to yield the proper configuration and fluorescent properties (Mares et al., 2011). This technology allows for a high-throughput assays for chaperone activity and its inhibitors (Mares et al., 2011).

Recent work using NMR, indicated that PDI is able to distinguish between unfolded, partly folded, and fully folded proteins. In these studies, it was observed that the dissociation constant (K_D_) for fully unfolded basic pancreatic trypsin inhibitor was ~1.5 μM (Irvine et al., 2014). On the other hand the fully folded protein had a K_D_ that was ~10 fold higher. Partially unfolded protein had a K_D_ that was ~3 fold higher. These data lead the authors to conclude that PDI can distinguish between unfolded, partially unfolded, and folded proteins (Irvine et al., 2014).

A major recent discovery shows that the chaperone activity of PDI is regulated by its redox status. Wang and coworkers were the first to obtain the crystal structure of human PDI in both the reduced and oxidized forms (Wang et al., 2012). In oxidized form of PDI the active site of ***a*** and ***a'*** are 40.3 Å apart and the thioredoxin domains ***abb'a'*** were all in the same plane (Wang et al., 2013). In the reduced state of PDI the active sites are 27.6 Å, however only ***abb'*** are in the same plane where ***a'*** is twisted 45° (Figure 2) (Wang et al., 2013), illustrating that the oxidized state has a more open conformation allowing for the entry of chaperone substrates (i.e., unfolded peptides) and the reduced state has a closed conformation inhibiting their entry. This further illustrates long range conformational changes induced by redox status of the active sites and further suggests redox regulation of chaperone activity.

Another interesting observation came from the work of Fu et al., where the hormone 17β-estradiol was able to bind to the hydrophobic cavity formed between the ***bb'*** domains (Fu et al., 2011). It should be noted that this site is different from the putative chaperone binding site in the ***b'*** domain. The key amino acid residue in the interaction of PDI with 17β-estradiol was shown to H256 where it is believed that nitrogen of the histidine forms a hydrogen bond with the 3-hydroxyl group (Fu et al., 2011). The authors, based on this data, postulate yet another physiological role for PDI: a reservoir for hormones (Fu et al., 2011).

## PDI redox activity and endoplasmic reticulum oxidoreductin-1

When PDI catalyzes the oxidation of thiols and the reduction and isomerization of disulfides, the catalytic vicinal active site thiols (-CXXC-) undergo sequential oxidation and reduction reactions. It is suggested that an oxidized ***a***-domain catalyzes the oxidation of the reduced substrates and in the process becomes reduced. The N-terminus cysteine (C53) on the catalytic motif reacts with the substrate to form the mixed heterodimer while the C-terminus cysteine (C56) releases the substrate (Walker and Gilbert, 1997). The ***a***-domain is then subsequently oxidized by the ***a'***-domain back to a disulfide through intramolecular reactions (Araki et al., 2013). The re-oxidation of the ***a'***-domain is catalyzed by the protein endoplasmic reticulum oxidoreductin-1 (Ero1) in the process reducing O_2_ to produce H_2_O_2_ (Araki and Nagata, 2011). It should be noted that this enzyme has two isoforms α and β (Wang et al., 2011). The binding affinity and catalytic activity of Ero1α with PDI was observed to be better at pH 7, and less so at 7.5 and 8 (Araki and Nagata, 2011). The interaction of Ero1α with PDI occurs through the interaction of the β hairpin in Ero1α with the ***b'***-domain of reduced PDI only (Masui et al., 2011). The non-covalent interaction was found to be between the aromatic residues, the W272 of Ero1α and F240, F249, and F304 of PDI (Masui et al., 2011). This interaction allows for Ero1α to be positioned in a manner that allows the oxidation of the ***a'***-domain of PDI but not the ***a***-domain (Araki and Nagata, 2011; Masui et al., 2011). NMR studies on Ero1α indicate that it is inactivated when disulfides are formed between C94–C104 and C99–C131 (Araki and Nagata, 2011). This conclusion was reached since C99 is the residue responsible for oxidizing the ***a'***-domain of PDI. Therefore, it is thought the formation of disulfide between C94 and C104 results in the C99 close C131. The subsequent formation of the disulfide C99–C131 thus inactivates Ero1α (Araki et al., 2013). The other isoform, Ero1β was observed to also preferentially oxidize the ***a'*** domain of PDI (Nguyen et al., 2011; Wang et al., 2011).

A further consequence of Ero1 activity is the oxidation of the ***a*-**domain of PDI. This is modulated through three other ER resident proteins: peroxiredoxin 4 (Prx IV), glutathione peroxidase 7 (GPx7), and glutathione peroxidase 8 (GPx8) (Kakihana et al., 2013; Wang et al., 2014). GPx7 and GPx8 catalyzes the reduction of Ero1-produced H_2_O_2_ to water. In the process, the C57 and C86 of GPx7 are converted to sulfenic acid and disulfide respectively. The PDI ***a***-domain then reduces these thiols, becoming oxidized in the process (Wang et al., 2014). When GPx7 or GPx8 was replaced with Prx IV this caused a slower oxidative folding of protein in comparison with either GPx7 or GPx8 (Nguyen et al., 2011). It is still controversial whether proteins such as, Ero1α, are the primary cause of the oxidation of PDI, since oxidized glutathione (GSSG) can also oxidize PDI active sites (Lappi and Ruddock, 2011). The second-order rate constant for the formation of mixed disulfide from reduced PDI and GSSG was observed to be 191 ± 3 M^−1^s^−1^ (Lappi and Ruddock, 2011). *In vivo* studies of the rate of oxidation of the ***a*** domain of PDI by GSSG were found to be ~0.3 s^−1^, indicating that this rate is too fast to be overlooked (Lappi and Ruddock, 2011). The oxidation of PDI by excess H_2_O_2_ is also observed and has a pseudo-first-order reaction with a rate constant of 9.2 M^−1^s^−1^ (Karala et al., 2009). However this study was conducted solely on the ***a*** domain on PDI, further studies of glutathione and glutathione disulfide on the oxidation of PDI are required to determine how much affect these molecules have on PDI. It should also be noted that more research must be conducted on how much affect PDI has on controlling the equilibrium of glutathione and glutathione disulfide.

## PDI inhibitors

With the importance of PDI in many different cellular functions, there has been an increased interest in the modulation of its activity with small molecule inhibitors. A commonly used inhibitor of PDI is bacitracin, which is a mixture of cyclic polypeptides. Recently, the analogs of bacitracin were individually tested to observe their inhibitory effect on the reductive activity of PDI. The analogs H and F were 25-fold more effective than the A and B analogs (Dickerhof et al., 2011). These analogs bind to the **b'** domain and interestingly do not seem to inhibit the oxidative and isomerase activity of PDI (Roth, 1981; Dickerhof et al., 2011).

Another inhibitor of PDI reductase activity is quercetin-3-rutinoside. This compound inhibits PDI mediated platelet aggregation as a result has been suggested as antithrombic agent (Jasuja et al., 2012). Phenyl vinyl sulfonate containing molecules were also showed to inhibit PDI (Ge et al., 2013). The inhibitor designated P1 was observed to inhibit PDI in mammalian cells with an IC_50_ of 1.7 ± 0.4 μm (Ge et al., 2013).

Propynoic acid carbamoyl methyl amides (PACMA) also inhibit PDI (Xu et al., 2012). Of these, the one designated PACMA31 was observed to be an irreversible inhibitor, by forming a covalent (carbon-sulfur) bond to the active site cysteine (Xu et al., 2012). Intriguingly, this inhibitor was observed to target ovarian tumors, inhibiting their growth but not causing harm to normal cells (Xu et al., 2012).

## PDI and NADPH oxidase

NADPH oxidase complex (Nox) is the major contributor of reactive oxygen species (ROS) in cells which can act as molecular signal and when produced in excess can cause oxidative stress (Janiszewski et al., 2005; Clempus and Griendling, 2006; Bedard and Krause, 2007; Marchetti et al., 2007). The NADPH oxidase complex is comprised of many subunits, that are expressed differently depending on the cell type (Janiszewski et al., 2005; Clempus and Griendling, 2006; Bedard and Krause, 2007; Marchetti et al., 2007). Laurindo's group were the first to report a role for PDI in the regulation of Nox. PDI is shown to interact with Nox within the ER as well as outside the ER in the cytosol (Laurindo et al., 2008, 2014; Santos et al., 2009b).

More recently, Fernandes et al. showed that in vascular smooth muscle cells, the overexpression of PDI increased Nox1 mRNA which encodes for NOX1 in NADPH oxidase complex (Fernandes et al., 2009). The PDI overexpression also produced an increase in the activity of NADPH oxidase, leading to an increase in the steady-state levels of ROS (Fernandes et al., 2009). Silencing of PDI triggered a decrease in mRNA and protein of Nox1 (Fernandes et al., 2009). Overexpression of a mutant PDI where the catalytic cysteines were mutated resulted in the increase in Nox1 mRNA and protein indicating that PDI redox activity is not required and it is most likely the chaperone activity of PDI that is modulating this effect (Fernandes et al., 2009).

In other studies, PDI silencing has shown to greatly decrease platelet derived growth factor (PDGF)-induced VSMC migration (Pescatore et al., 2012). In the same study, it was observed that PDI co-immunoprecipitated with Rac1, RhoA, and RhoGDI (Pescatore et al., 2012). In macrophages, PDI plays a similar role where interaction with NADPH oxidase causes an increase in ROS (Santos et al., 2009a). Laurindo's group has shown that PDI co-immuneprecipitated and co-localized with p22phox subunit of NADPH oxidase in J774 cells (Santos et al., 2009a). They also used a leukocyte cell free system to observe the interaction of PDI with NADPH oxidase. They found that oxidized PDI stimulates ROS production however reduced PDI inhibited the production of ROS (De et al., 2011). This group further suggests that PDI possibly interacts with p47phox subunit of NADPH oxidase through hydrophobic effects and not through cysteines (Santos et al., 2009a). From all this data it can be suggested that PDI plays an important role in the regulation of ROS.

## PDI and infection

The internalization of some pathogens has been shown to be modulated by PDI. Ryser and coworkers were the first to test the potential role of PDI in the internalization of pathogens (Ryser et al., 1994). In mouse macrophage J774 cells, Laurindo's group observed that increased levels of PDI would increase the phagocytosis of *L. chagasi* promastigote but not the amastigote (Santos et al., 2009a). Phagocytosis was decreased by inhibition of expression levels of PDI as well as through the addition of thiol inhibitors such as *p*-phenylarsine oxide (Santos et al., 2009a). Santos and coworkers hypothesized that PDI plays a role in the reduction of the disulfide bonds present on the parasite, which may help with internalization of the parasite (Santos et al., 2009a). They also suggest that PDI's role in the increase of ROS through the NADPH oxidase interaction resulting in an “intraphagosomal-oxidizing milieu” and thus favoring infection of promastigotes (Santos et al., 2009a).

Reiser and coworkers have examined the role of PDI in HIV infections. Both PDI and thioredoxin 1 (Trx1) have been shown to reduce disulfides on the viral glycoprotein gp120 causing the internalization of HIV-1 (Reiser et al., 2012). Through semi-quantitative ELISA analysis it was observed that Trx1 was more efficient at reducing disulfides on gp120, however the authors hypothesize that the two proteins may be acting at different stages of internalization (Reiser et al., 2012). Interestingly, galectin-9 was observed to bind to PDI on Th2 cells this helped with the retention of PDI to the cell surface as well as increased the entry of HIV into these cells (Bi et al., 2011). It is suggested that inhibitors of gp120 that prevent the reduction of the disulfides maybe a better drug target than reductases since reductases are widely used in cells (Reiser et al., 2012).

In endothelial cells surface PDI possibly reduces β1 and β3 integrins allowing for the entry of dengue virus (Wan et al., 2012). Also in MA104 cells thiol blockers and PDI inhibitors decreased the entry of rotavirus, indicating the involvement of thiols for infectivity (Calderon et al., 2012).

Recently, PDI was observed to regulate the cytoskeleton reorganization by its interaction with β-actin (Sobierajska et al., 2014). PDI was observed to bind to Cys374 of β-actin through a disulfide bond (Sobierajska et al., 2014). In MEG-01 cells the down regulation of PDI resulted in the prevention of adhesion to fibronectin (Sobierajska et al., 2014).

During the bacterial infection of the cholera toxin it is observed that PDI does not unfold the CT protein into its subunit CTA1 for further infection (Taylor et al., 2011). Nevertheless, PDI does disassemble the holotoxin allowing for the dissociation of CTA1 from CTA2 and CTB_5_ and its spontaneous unfolding, allowing CTA1 to exit the ER (Taylor et al., 2011). Interestingly, Taylor and coworkers recently discovered that PDI when interacting with the holotoxin, will itself unfold, causing a wedge to form between the holotoxin and causing the dissociation of CTA1 (Taylor et al., 2014). PDI locked in the folded state would not dissociate the holotoxin (Taylor et al., 2014). This appears not to required the oxireductase activity of PDI since redox inhibitors had no effect on toxin activation. On the other hand, chaperone inhibitors prevent the release of CTA1 thus indicating that PDI unfolding in the presence of holotoxin requires PDI chaperone activity (Taylor et al., 2014).

## PDI and coagulation

The activation of platelets is a complex series of proteolytic, protein-protein, and protein-ligand interactions that is not yet fully understood. A quick summary of this process is, that damaged endothelial cells will cause the exposure of collagen allowing for the glycoprotein, von Willebrand factor to attract platelets (Lopez et al., 1992; Modderman et al., 1992; Ruggeri, 2007; Herr and Farndale, 2009). The platelets form a monolayer, which in turns produces thrombin for additional platelet aggregation and causes a series of cascades (Monroe et al., 2002; Clemetson, 2012). The platelets also start to secrete other activators these will bind to receptor on the platelets and increase the intracellular calcium levels (Purvis et al., 2008). This causes receptors on platelets to bind fibrinogen resulting in the increase adhesion of the platelets and the formation of thrombi(Ma et al., 2007; Furie and Furie, 2008). Many of the integrin receptors associated with platelet activation contain a cysteine rich subunit, possibly allowing for PDI to regulate the redox state of the thiols.

Platelets deficient of PDI display irregular and improper aggregation, when wild-type PDI is added the aggregation of platelets returns to normal (Kim et al., 2013). PDI on the surface of platelets plays a crucial role in the formation of thrombus in collagen-coated platelets (Kim et al., 2013). In monocytic cells, cell surface PDI is required for antithymocyte globulin decryption of tissue factor (Langer et al., 2013).

Recent studies have shown that free thiols and isomerization are associated with the process of coagulation (Jurk et al., 2011). *In vitro* studies by Jurk and coworkers have shown that PDI plays a role in the feedback activation of thrombin in platelets that have already been stimulated by thrombin (Jurk et al., 2011). In the same paper, they showed that PDI may play a role in the interaction of coagulation factors with platelets (Jurk et al., 2011). With the use of CHO cells expressing different integrin factors, α_V_β_3_ and α_IIb_β_3_, PDI was observed to bind to these integrin molecules (Cho et al., 2012). This binding of PDI with the integrin was determined to be through the β_3_ subunit and not the α subunit (Cho et al., 2012). The binding of PDI to integrins was not inhibited by the presence of PDI redox inhibitors, the authors however do not hypothesize how this interaction is occurring (Cho et al., 2012). In a mice model, where there is a deficiency in β_3_ integrins, it is observed that fibrin generation was diminished from the lack of accumulation of PDI at the platelet surface (Cho et al., 2012). In neutrophil cells, PDI may mediate the adhesive activity during vasculature inflammation through its interaction with α_M_β_2_ integrin (Hahm et al., 2013). With the use of flow cytometry, it was observed that exogenous PDI interacted with neutrophil cell surface by electrostatic interactions (Hahm et al., 2013). The authors propose that PDI catalyzes thiol exchange through electrostatic interactions of α_M_β_2_ integrin thus regulating the clustering of the integrin within lipid rafts (Hahm et al., 2013). It should also be noted that vitamin K epoxide reductase, an important enzyme in blood coagulation, requires the help of a thioredoxin-like protein to regenerate vitamin K hydroquinone from the epoxide form. It is speculated that the PDI is the thioredoxin-like protein that provides the reductive equivalent to perform the regeneration (Schulman et al., 2010).

In endothelial cells, when PDI was silenced or inhibited on the cell surface, the coagulation was increased (Popescu et al., 2010). In agreement with this finding, it was observed that addition of exogenous PDI resulted in the decrease in coagulation activity (Popescu et al., 2010). The authors suggest that coagulation of endothelial cells is negatively regulated by PDI, specifically its oxidoreductase activity (Popescu et al., 2010). In the same study, it was also observed that PDI may play a role in the regulation of phosphatidylserine exposure through the activity of flippase and floppase (Popescu et al., 2010).

For more information of previous knowledge on the effects of redox on coagulation can be found in reviews by Cho (2013), Flaumenhaft (2013), Langer and Ruf (2014), and Murphy et al. (2014).

## PDI and superoxide dismutase 1

Protein aggregation has been shown to occur in a variety of neurodegenerative diseases and disorders, such as cerebral ischemia and amyotrophic lateral sclerosis. Cai and coworkers were one of the first to show that PDI alleviates the aggregation of proteins (Cai et al., 1994). Cerebral ischemia is caused by decrease blood flow to the brain resulting in the loss of oxygen to neurons and astrocytes, which has shown to cause an increase in nitric oxide (NO) production specifically by inducible nitric oxide synthase (iNOS) (Cherian et al., 2000; Greve and Zink, 2009). Elevated NO production can lead to the inactivation of proteins by *S*-nitrosylation. In astrocytes, recent studies by Chen have shown the presence of S-nitrosylated PDI (SNO-PDI), which has previously been shown to inhibit the activity of PDI (Ramachandran et al., 2001; Chen et al., 2012). When the cells were oxygen deprived, this caused the formation of ubiquitinated protein aggregates, of proteins like superoxide dismutase 1 (SOD1), which is crucial to cell survival (Chen et al., 2012).

When NO production in these cells was attenuated, the formation of both SNO-PDI and protein aggregates decreased (Chen et al., 2012). Interestingly, in SH-SY5Y cells the overexpression of mutant SOD1 caused an increase expression of iNOS, where wild type SOD1 did not cause any increase (Chen et al., 2013). SOD1 is a protein that alleviates oxidative stress by converting free radicals to H_2_O_2_ (Chen et al., 2012). The mutant SOD1 expressing cells were observed to contain SNO-PDI and protein aggregation while the wild type SOD1 did not show any increase in either, this was also observed *in vivo*, in transgenic mice (Chen et al., 2013). Furthermore, the expression of PDI increased when protein aggregates accumulated in cells, and the cells became apoptotic (Hoffstrom et al., 2010; Chen et al., 2013).

The aggregation of mutant SOD1 has shown to cause an increase in neuronal cell death in amyotrophic lateral sclerosis, it has been hypothesized that denitrosylation of PDI can be considered as a therapeutic intervention for this disease (Jeon et al., 2014). In both HEK293A cells and NSC-34 motor neuron cells, the overexpression of PDI lead to a decrease in the aggregation of mutant SOD1 (Jeon et al., 2014). The interaction of PDI with SOD1 appears to be through the chaperone activity of PDI rather than the isomerase activity since aggregation was also reduced with mutant PDI lacking active site thiols (Jeon et al., 2014). Intriguingly, the overexpression of PDI caused by mutant SOD1 has shown to cause the activation of NADPH oxidase causing an increase in ROS (Jaronen et al., 2013). In murine microglial BV-2 cells Nox activation was PDI-dependent suggesting that PDI inhibitors may help reduce oxidative stress in cells (Jaronen et al., 2013). However, further studies must be conducted to determine PDI's role in the interplay of protein aggregation and the production of ROS.

## Role of PDI in nitric oxide signaling

Our group was the first to show that cell surface PDI mediated the entry of *S*-nitrosothiols (SNO) into cells (Ramachandran et al., 2001). Cell surface PDI was observed to release NO from *S*-nitrosylated glutathione (GSNO), one of the main nitric oxide carriers in tissues (Root et al., 2004). PDI would denitrosylate GSNO when NO levels were low, however during high levels of NO, PDI would act as a carrier either through the formation of SNO-PDI (Sliskovic et al., 2005).

**Figure 3:**
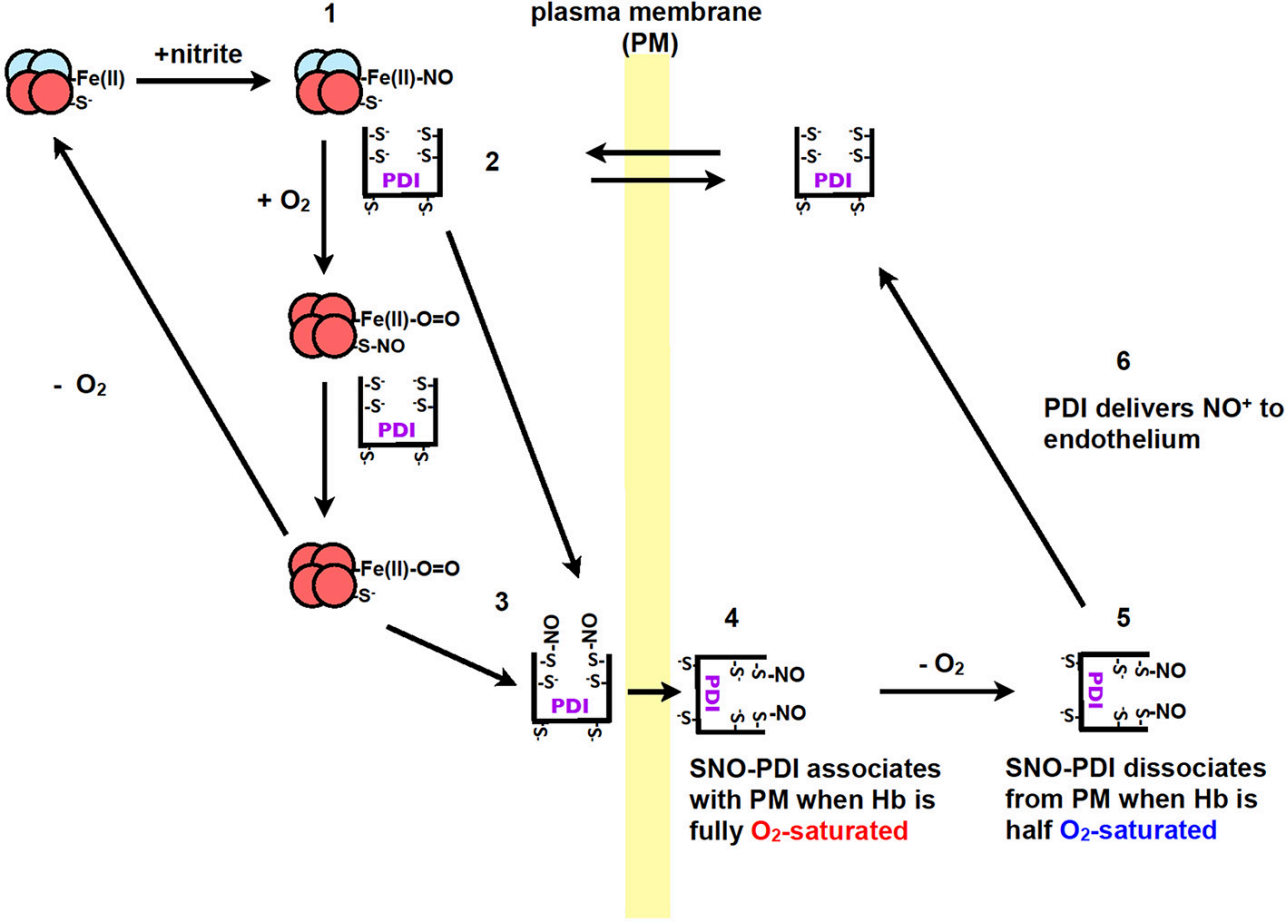
**The formation of NO^+^/NO from nitrite during hypoxic conditions by Hb and the transfer of nitric oxide to endothelium by PDI**. (1) Nitrite reacts with Hb under hypoxic conditions to form Fe(II)-NO. (2) PDI from blood equilibrates across the plasma membrane of red blood cells to form a complex with Hb. (3) When red blood cells enter the lungs the O_2_ displaces the NO from the iron on the heme group to PDI thiols or to Hb (Cys β93), resulting in the formation of SNO-PDI. (4) PDI attaches to the extracellular surface of red blood cells under normoxic conditions. (5) When the red blood cells enter tissue that is under hypoxic conditions, SNO-PDI is released. (6) SNO-PDI interacts with the endothelium cells, releasing NO^+^/NO triggering hypoxic vasodilation. Image taken from Kallakunta et al. (2013).

Recently our group, showed that PDI plays a role in the transport of nitric oxide from red blood cells to endothelium cells (Kallakunta et al., 2013). This process was observed to be oxygen-dependent where nitric oxide bound hemoglobin (NO-Hb) required hypoxic conditions. Oxygen is then required for the formation of SNO-Hb, which then transfers the nitric oxide to PDI to form SNO-PDI (Kallakunta et al., 2013). When hemoglobin is half oxygen saturated it was observed that nitric oxide would dissociate from extracellular PDI and be transferred to endothelium cells (Figure 3) (Kallakunta et al., 2013).

## Conclusion

During the early years of research on PDI it was well thought of that PDI was required for the maintenance of healthy cells and tissues, in view of the catalytic roles of PDI in reducing and isomerizing disulfides into native confirmations of proteins and to alleviate protein aggregates through its chaperone activity. However recent studies indicate that this may not be the case. PDI through its thiol redox activity, is observed to play a role for the internalization of some pathogens, as well as it is shown to promote increased ROS in cells. Furthermore, in neuronal cells post transnationally modified SNO-PDI etc. has been shown to promote protein aggregation commonly associated with the pathophysiology of neurodegenerative diseases. These observations show that PDI is a complex protein that can play a role in both physiology and pathophysiology. Therefore, further studies are warranted in order to solve the complex relationships of PDI in health and disease.

### Conflict of interest statement

The authors declare that the research was conducted in the absence of any commercial or financial relationships that could be construed as a potential conflict of interest.
